# The development and implementation of a training package for dietitians on cow’s milk protein allergy in infants and children based on UK RCPCH competencies for food allergies – a pilot study

**DOI:** 10.1186/s13601-015-0046-y

**Published:** 2015-02-03

**Authors:** Liane Reeves, Rosan Meyer, Judith Holloway, Carina Venter

**Affiliations:** Community Nutrition & Dietetic Department, East Oxford Health Centre, Cowley Road, Oxford, OX4 1XD UK; MSc Allergy, University of Southampton, Southampton General Hospital, Tremona Road, Southampton, SO16 6YD UK; Department of Gastroenterology, Great Ormond Street Hospital for Sick Children, London, WC1N 3JH UK; University of Portsmouth, James Watson West, 2 King Richard 1st Road, Portsmouth, PO1 2FR UK; The David Hide Asthma and Allergy Research Centre, St Mary’s Hospital, Newport, Isle of Wight PO30 5TG UK

**Keywords:** Competencies, Cow’s milk protein allergy, Dietitian, Training

## Abstract

**Background:**

Many food allergy guidelines have been published worldwide over recent years. The United Kingdom National Institute of Health and Clinical Excellence guidelines and The Royal College of Paediatrics and Child Health food allergy care pathways require dietitians to assist with the diagnosis and management of food allergies, which highlighted the need for further education of dietitians to meet these competencies. The aim of this study was to design a competence based one day education course for dietitians on the diagnosis and management of cow’s milk protein allergy in infants and children.

**Methods:**

A one day training course was developed. Dietitians’ knowledge was assessed via multiple choice questions before and on the day of the course and retention of knowledge was assessed one month after the course. Pre course reading was given once the first assessment was completed.

**Results:**

Thirty seven dietitians attended the course and 32 completed all three assessments. A significant improvement in assessment scores was seen between the pre course and on the day assessments of 7.2% (p < 0.001) and between pre course and post course assessments of 8.9% (p < 0.001). In delegates who rated their perceived level of knowledge as *high,* a significant increase was seen between pre course and on the day and between pre course and post course (both p < 0.001). Actual increase in knowledge was seen alongside a significant increase in *high* rating of perceived level of confidence between pre course and on the day and between pre course and post course (both p < 0.001).

**Conclusions:**

Educating dietitians using the format of one day teaching with pre and post course assessment has improved both knowledge and competencies in the diagnosis and management of cow’s milk protein allergy. Further courses in other areas of food allergy could be developed using this approach within the UK and worldwide.

## Background

A number of food allergy guidelines have been published internationally in the past few years aimed at improving both the diagnosis and management of food allergies. These include the World Allergy Organisation (WAO) Diagnosis and Rationale for Action against Cow’s Milk Allergy (DRACMA) [1], the United States’ National Institute of Allergy and Infectious Diseases (NIAID) guidelines [2], the United Kingdom (UK) National Institute of Health and Clinical Excellence (NICE) guidelines [3] and the European Society for Paediatric Gastroenterology, Hepatology and Nutrition (ESPGHAN) guidelines on the diagnostic approach and management of cow’s milk allergy in 2012 [4]. More recently the European Academy of Allergy and Clinical Immunology (EAACI) also produced guidelines on the diagnosis and management of food allergy [5]. The Royal College of Paediatrics and Child Health (RCPCH) in the UK followed up the NICE guideline with allergy care pathways for children not only on the diagnostic process, but also on management with defined competencies [6]. Both these UK guideline documents recommend that health professionals, including dietitians, meet competencies in order to identify food allergies and provide optimal management strategies.

Although the majority of guidelines suggest that dietitians are ideally placed to give nutritional advice, the NICE food allergy guideline [3] is the only document recommending that dietitians with the relevant competencies should provide nutritional advice. In particular, competent dietitians, as defined by the RCPCH, may help with the choice of appropriate hypoallergenic formulas/milk alternatives in cow’s milk protein allergy (CMPA). The UK Milk Allergy in Primary Care (MAP) Guideline [7] also highlights how dietitians can provide guidance on milk avoidance and suitable alternative foods to achieve adequate nutritional status, as well as performing challenges for the initial diagnosis of CMPA and to determine development of tolerance. Adequate allergy training for dietitians is therefore vital for the effective management of the CMPA patient.

The level of general dietetic training varies between countries and the UK is the only country to offer MSc level training in allergy to dietitians (www.southampton.ac.uk/medicine/allergy and www1.imperial.ac.uk/departmentofmedicine/postgraduate/allergyprogramme/). Dietitians’ allergy knowledge therefore varies within and between countries. Surveys of dietitians in South Africa [8], the United States [9], the UK and Australia [10,11] found that further training was needed for dietitians in the management of food allergies. Specifically, perceived knowledge of diagnosing food allergy and developing food challenge protocols tended to be rated as moderate or low in the Australian and United States surveys whilst the majority of respondents rated their knowledge of the definitions, clinical features and dietetic management as either moderate or high [12]. A survey of UK dietitians’ learning needs in relation to food allergies and intolerances found that 39% felt they needed further training on CMPA. Sixty one percent preferred face to face combined with web based learning and 68% preferred training to be undertaken in less than 2 days [10]. We have used information from this UK survey to design our one-day educational course. Financial burdens in healthcare along with time pressures have lead to the need to develop affordable and easily accessible training courses for dietitians.

In light of international guidelines and dietitians’ perception of needing to improve level of knowledge, we set out to design a pilot course for dietitians based on national competencies [6]. The course focused on the diagnosis and management of CMPA in infants and children and intended to provide a ‘step-up’ from the traditional one day courses to enable dietitians to show competency in the management of this allergy. The course was supported by the Food Allergy and Intolerance Specialist Group (FAISG) of the British Dietetic Association (BDA) (www.bda.uk.com/regionsgroups/groups/foodallergy/home).

## Methods

Ethical approval was not required.

### Course development and execution

#### Assessment questions

The definition of ‘competence’ for this study denotes being able to diagnose and manage patients with allergies safely and effectively as per NICE guidelines [3]. This definition was kept in mind when developing the assessment tool. Twenty-eight multiple choice questions were designed to relate to each objective and competence. Questions were a combination of fact based e.g. ‘what is the best definition of…..’ and case scenarios in design. All questions required the participant to choose one best answer and were marked out of a possible score of 31 as one question contained 4 parts.

The assessment questions were developed by comparing the knowledge of two groups of volunteer dietitians recruited via emailing FAISG members; 10 with little or no experience in allergy and 10 who were experienced allergy dietitians. The assessment was delivered online via www.surveymonkey.co.uk and the dietitians were given the opportunity to answer and comment on each question via this electronic form. Once completed, responses were reviewed and the wording of some questions adjusted to clarify or adjust the difficulty of the question.

#### Pre course reading

Delegates were asked to read the NICE food allergy quick reference guide [13].

#### Course format and assessment

Presentations were designed to meet the course objectives outlined in Table 1 and speakers were selected according to their experience in the diagnosis and management of CMPA.

**Table 1:** **Objectives for each session**

**Timing & Topic**	**Competences/objectives**	
	**Once completed, dietitians should:**	
	**Know**	**Be able to:**
**9.30-10.00 Background to allergy**	• the major categories of adverse reactions to foods	• recognise that food allergy may present in a variety of ways ranging from immediate allergic reactions to more chronic presentations such as eczema or gastro-intestinal symptoms
	• that food allergy may present in a variety of ways	
	• that many common childhood conditions e.g. eczema, gastro-oesophageal reflux may have an allergic aetiology	
	• that food allergy is more common in children with early onset eczema, particularly mild to moderate eczema	• recognise the risk factors for allergic aetiology of presenting features such as family or personal history of atopy
	• the common foods which are responsible for most food allergies in children	• differentiate different types of adverse reactions to food based on findings from history and examination
**10.00-11.15 Diagnosis & interpreting tests**	• that the level of sIgE varies and should not be used in place of oral food challenges to determine allergy e.g. cow’s milk	• take and interpret an allergy focused clinical history
	• that skin prick tests and sIgE have a poor predictive value for non-IgE mediated allergies	• differentiate different types of adverse reactions to food based on findings from the history
	• that atopy patch tests are available but that their role in the diagnosis of food allergy remains unclear	• gather information on relevant exposures to other potential food allergens and take a dietary history including the interpretation of a food and symptom diary
	• that complementary and alternative medicine allergy tests, including kinesiology, serum sIgG and vega tests have no place in the diagnosis and/or management of food allergy	• interpret SPT results in the context of the clinical history
		• interpret serum sIgE results in the context of the clinical history
**11.30-12.45 Diagnostic diets & food challenges**	• which diagnostic diet is appropriate to use according to symptoms	• advise about the safe reintroduction of cow’s milk following a negative food challenge
	• which formulas are available for managing CMP allergy and lactose intolerance	• recommend an appropriate formula according to symptoms and clinical history
	• which oral challenges may be done as open challenges, which need medical supervision and which are suitable for home	
	• when it is appropriate to challenge and how to decide on challenge outcome	
**13.30-14.30 Dietary management**	• what foods (including catering, manufactured ingredients and manufactured foods) are likely to contain trigger foods	• advise on appropriate dietary exclusion and alternatives including practical individualised advice (e.g. appropriate to age, culture etc. )
	• clinically relevant cross-reactivities	• educate patients, parents and carers about effective food allergen avoidance including high risk situations e.g. eating out
	• common situations when allergen exposure is most likely to occur (e.g. eating out)	• advise patients, parents and carers of issues relating to risk in specific situations e.g. school
	• the risks inherent to specific situations (e.g. home, school, eating out and hospital settings)	• provide support to patients and families to help minimise the impact of food allergy on quality of life through education, ongoing access and patient queries
**14.30-15.30 Nutritional issues & weaning**	• how to recognise that faltering growth is a result of food allergy	• give practical advice on weaning the cow’s milk allergic infant
	• when it is appropriate to refer to other health care professionals	• provide details of resources including patient charities, websites and local support groups
		• ensure the nutritional requirements of infants and children on a CMP free diet are met
		• manage nutritional deficiencies and faltering growth
**15.45-17.00**	**Case studies discussion and final assessment**	

CMP; cow’s milk protein, sIgE; specific IgE, sIgG; specific IgG, SPT; skin prick tests.

Delegates were recruited via advertisement in the BDA magazine and website and contacted the BDA to book a place. Places were charged at the standard BDA rate of £95. Delegates were asked to complete online assessment questions prior to the course (pre course assessment) using their unique number given at registration in order to remain anonymous for analysis. Assessment questions were completed again at the end of the day on paper, and online a month after the course (post course assessment) in order to measure whether knowledge had been retained. Based on clinical competencies, achieving ≥90% in the post course assessment questions was considered sufficient to pass the course and receive a competence certificate. Delegates were informed of the criteria prior to the course and the reason for completing assessments at each stage. They were also asked to complete a general evaluation at the end of the day to assess speakers, venue and overall satisfaction with the course.

At each assessment, delegates were asked to state their perceived level of knowledge of allergies on a Likert scale of none, a little, a fair amount or a lot. They were also asked to rate their level of confidence in managing children with CMPA on a Likert scale of 1 (not at all confident) to 5 (very confident). Following completion of the post course assessment, all delegates were asked to answer one online question to indicate whether they had completed any of the recommended reading prior to answering the post course questions.

**Figure 1:**
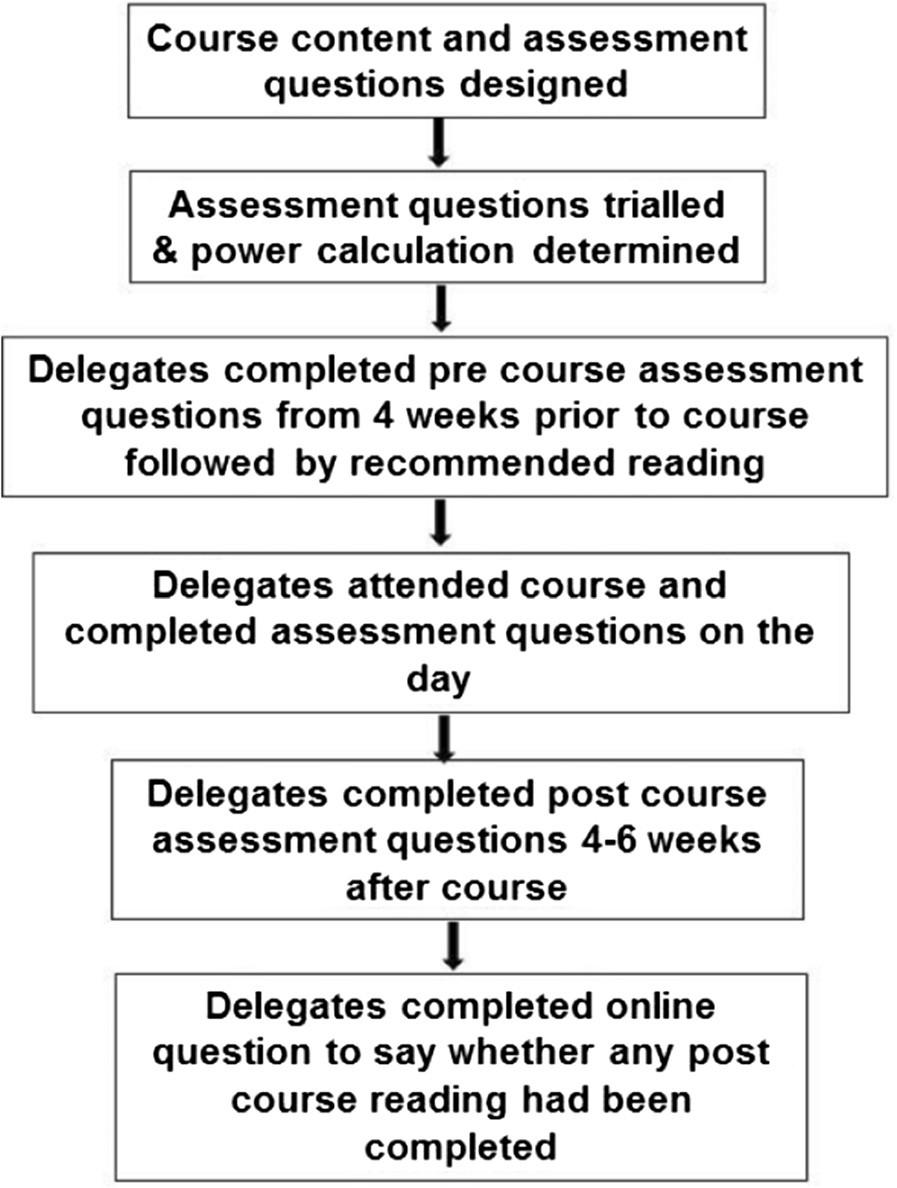
**Process of designing and implementing course.**

A summary of the process of designing and implementing the course can be found in Figure 1.

### Statistical analysis

#### Power calculation

Results from the 20 volunteer dietitians who trialled the questions were assessed. Seven dietitians identified themselves as being proficient in the management of food allergy and these were compared to 7 dietitians with no experience in managing food allergies. The remaining six dietitians stated that they had a little allergy management knowledge and were not included in the calculation. Mean scores were 26.28 (out of 31) in the experienced group and 19.85 in the non-experienced group (effect size 6.43 and SD 4.2). Based on these results a power calculation determined that with 13 delegates in each group, or 26 in total, we would be able to detect a significant difference between pre and post assessment scores at a significance level of 0.01 and 90% power. It was agreed that we would aim for 30 delegates to allow for any drop-outs.

Statistical analyses were performed using SPSS version 19 for Windows (IBM, New York, USA). The Wilcoxon signed rank test was used to determine whether there were significant differences between assessment scores and perceived level of confidence between assessments. The McNemar test was used to determine whether there was a significant change in perceived level of knowledge between assessments and the Fishers exact test was used to determine the relationship between previous training and whether delegates passed or failed.

The Chi-square test was used to investigate the relationship between the NHS grade delegates were employed at (and therefore their level of experience) and passing or failing the post course assessment. A p value of ≤ 0.05 was considered to be statistically significant.

## Results

### Subjects

Thirty seven dietitians attended the course. Thirty two delegates completed all 3 assessments and results from these candidates were compared. Table 2 illustrates the delegates’ characteristics.

**Table 2:** **Characteristics of delegates who completed pre course, on the day and post course assessments**

**Practice Setting (N = 32)**	**N**	**%**
**(delegates could choose more than one)**		
*Hospital outpatients*	12	37.5
*Hospital inpatients*	17	53
*Community*	14	44
*Private Practice*	4	12.5
*Other (does not practice/industry)*	1	3
**Current Post NHS Band**		
*5 (Basic Grade)*	8	25
*6 (Specialist)*	15	47
*7 (Advanced)*	5	16
*Not given*	4	12
**Type of patients usually seen**		
*Paediatric*	13	41
*Adult*	4	12
*Both paediatric and adult*	14	44
*Not given*	1	3
**Previous training in allergy**		
**(delegates could choose more than one)**		
*No previous allergy training*	17	53
*Specialised one day food allergy conferences*	9	28
*BDA Paediatric Course*	4	12
*Post graduate cert/dip/MSc*	1	3
*Other:*		
*Shadowing experienced dietitians*	3	9
*Not given*	2	6

BDA; British Dietetic Association, N; number of delegates.

### Assessment results

The assessments were marked out of a possible 27 points after 4 questions were removed due to the subject matter not being covered adequately. Twenty four (75%) delegates achieved a pass rate of ≥90% on the post course assessments. Eight (25%) delegates therefore did not pass the post course assessment. Mean results for the pre-course, on the day and post course assessments were 84%, 91% and 93% respectively.

A significant improvement in scores was seen between the pre course assessments and on the day assessments of 7.2% (p < 0.001) and between the pre course assessments and post course assessments of 8.9% (p < 0.001). However there was no significant change in the scores between the on the day assessment and post course assessment, indicating that the information had been retained by the delegates (p = 0.175).

Twenty (62.5%) delegates completed some recommended reading after the course. Of the 24 delegates who passed, 12 (50%) had read some of the recommended reading and 2 (8%) had read all.

### Perceived level of knowledge in managing allergies

**Figure 2:**
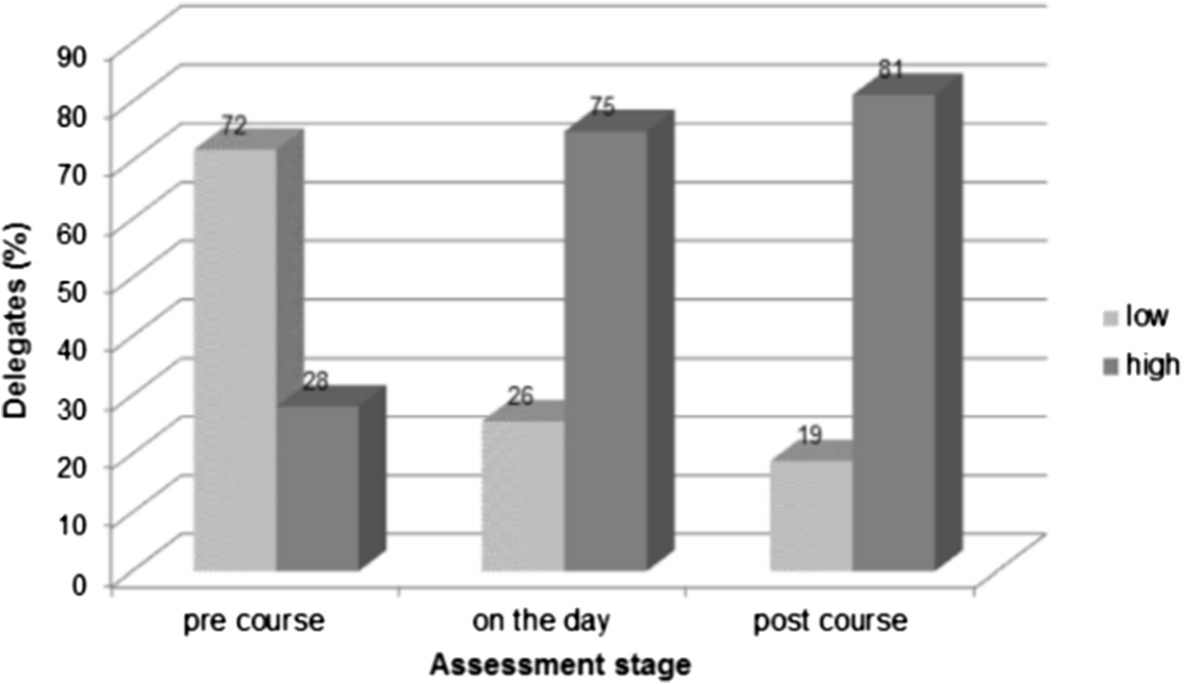
**Perceived level of knowledge at pre course, on the day and post course assessments**.

A significant difference in delegates rating their perceived level of knowledge as high was seen between pre course and on the day (p < 0.001) and pre course and post course (p < 0.001). The increase between the day of the course and post course perceived knowledge was not significant (p = 0.5). Figure 2 shows the change in perceived level of knowledge of allergies at each assessment.

### Perceived level of confidence in managing CMPA

**Figure 3:**
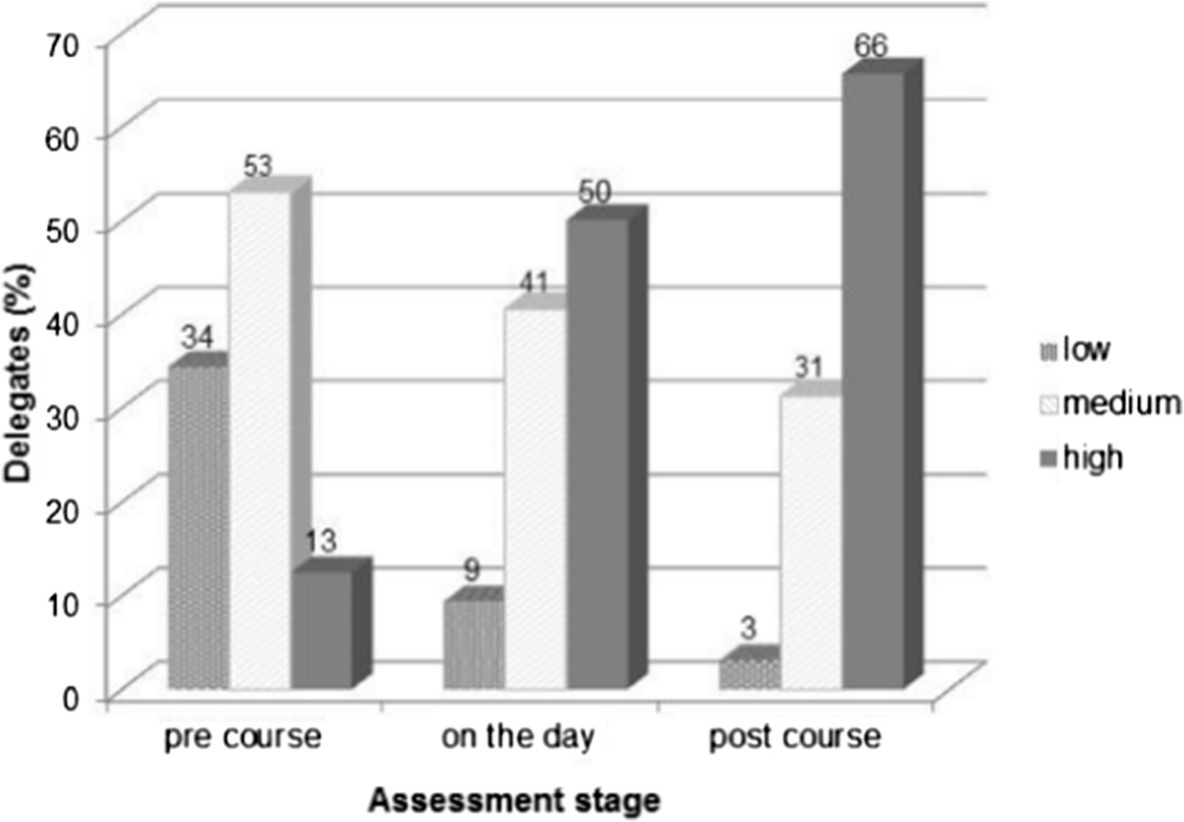
**Perceived level of confidence in managing CMPA at pre course, on the day and post course assessments.**

A significant increase in delegates rating their perceived level of confidence as high in managing children with CMPA was seen between pre course and on the day (p < 0.001), on the day to post course (p = 0.008) and between pre course and post course (p < 0.001). Figure 3 shows the change in perceived level of confidence in managing CMPA at each assessment.

### Previous training

Thirty (94%) delegates gave information on previous food allergy related training they had attended. Of those that failed the post course assessment, 6 (75%) had no previous training in food allergy, 1 (12.5%) had a post graduate qualification in allergy and 1 (12.5%) had shadowed other dietitians.

Of those that passed the post course assessment, 11 (50%) had no previous training in food allergy, 5 (23%) had attended specialised one day food allergy conferences, 4 (18%) had attended both specialised one day food allergy conferences and the BDA paediatric course, and 2 (9%) had shadowed colleagues.

There was no significant relationship between the amount of previous training and whether delegates passed or failed (p = 0.221). There was a trend for the more skilled dietitians employed at higher NHS bands to have a higher pass rate (p = 0.038).

### Assessment questions

Table 3 shows the number of correct answers given for each question at each assessment. The biggest improvements between pre course and post course assessments were seen in questions relating to: eczema and food allergy (35%), symptoms (25%), milk ingredients (28.5%) and sIgE (22%).

**Table 3:** **Number (percentage) of correct answers given for each question on each assessment**

**Question topic**	**Pre course correct**	**On the day correct**	**Post course correct**
	**N (%)**	**N (%)**	**N (%)**
**Allergy definition**	26(81)	31(97)	31(97)
**Anaphylaxis**	32(100)	31(97)	32(100)
**Eczema & food allergy**	32(100)	30(94)	32(100)
**Eczema & food allergy**	18(56)	17(53)	29(91)
**Symptoms**	23(72)	31(97)	31(97)
**Allergy vs intolerance**	28(87.5)	32(100)	30(94)
**Milk formulae**	29(91)	31(97)	32(100)
**Milk formulae**	26(81)	28(87.5)	31(97)
**Milk formulae**	25(78)	28(87.5)	31(97)
**Milk formulae**	29(91)	32(100)	32(100)
**Which formula to recommend (case scenario)**	30(94)	30(94)	31(97)
**Which formula to recommend (case scenario)**	18(56)	25(78)	21(66)
**Which formula to recommend (case scenario)**	26(81)	28(87.5)	29(91)
**Diagnosis**	28(87.5)	32(100)	32(100)
**Skin prick testing**	25(78)	28(87.5)	27(84)
**Specific IgE**	15(47)	21(66)	22(69)
**Specific IgE**	28(87.5)	23(72)	24(75)
**Food challenges**	32(100)	32(100)	32(100)
**Cross reactions**	28(87.5)	31(97)	32(100)
**Cross contamination**	25(78)	32(100)	32(100)
**Milk ingredients**	20(62.5)	29(91)	29(91)
**Managing CMPA in schools**	(32(100)	32(100)	32(100)
**Appropriate resources**	32(100)	31(97)	30(94)
**Food challenging**	28(87.5)	32(100)	32(100)
**Food challenging**	31(97)	30(94)	31(97)
**Hidden sources of milk**	32(100)	32(100)	32(100)
**When to refer on**	26(81)	27(84)	27(84)

Two questions showed a decrease in correct answers between pre and post course assessments; on sIgE (12.5%) and on appropriate resources (6%).

Three questions were answered correctly by all delegates in all three assessments on: food challenges, managing CMPA in schools and hidden sources of milk.

### Evaluation of the course

All delegates gave a score of 8 or more out of 10 on the evaluation forms for enjoyment of the event, met expectations and resources available to take away.

## Discussion

The NICE food allergy guidelines and The RCPCH food allergy care pathway [3,6] were designed to improve knowledge and competencies of health professionals managing children with food allergies in order to improve patient care. We designed a one day course for dietitians consisting of 5 lectures and case study discussion that based session objectives on these national competencies. We have shown that dietitians’ knowledge of CMPA can be improved and retained by the delivery of a course in this format. Actual level of knowledge, perceived level of confidence and perceived knowledge all improved and knowledge was retained after one month. Satisfaction with the course was high and 75% achieved the required ≥90% to pass the post course assessment.

To our knowledge this is the first study looking at food allergy education for dietitians based on competencies, focusing on CMPA. The format was based on a survey of dietitians’ learning needs in the area of food allergies and intolerances [10]. Other studies have looked at food allergy education for physicians; Springston et al. [14] designed an online ‘Food Allergy Comprehension Tool’ (FACT) which consisted of multiple choice questions (MCQs) and case studies. Their brief education tool significantly improved knowledge in some of the areas addressed, however this did not involve any face to face education and was not based on competencies as in our study. The WAO have produced online learning modules [15] on different allergy topics which can be accessed by any health care professionals, but are not particularly tailored towards the needs of dietitians. They also use case scenarios to educate the participant but there is no a formal method of assessment to show that participants have met designated competencies.

Yu et al. [16] designed a one hour food allergy education programme for primary care physicians caring for teenagers and adults in the US. They based their program on lectures and a practical demonstration in a similar way to ours although much shorter in length. That study showed that knowledge could be improved using this approach. An intensive ‘boot camp’ format for residents and fellows in training is described by Elizalde et al. [17], where knowledge was assessed before and after the education sessions for six participants. Although they had a small sample size, a significant improvement in knowledge was shown from educating this group in this way. Similarly Swan et al. [18] also showed that knowledge could be improved by the delivery of a one day study day to health professionals by the assessment of knowledge before and at the end of the day.

However the strength of our study remains the proven retention of knowledge, which all the above quoted studies did not perform [14,16,17].

Our study included multiple choice questions marked out of a possible 27 points designed to assess the competencies linked to CMPA, which were completed before, at the end of the study day and repeated again 1 month after. Swan et al. [18] based their assessment on only 7 questions and used audience response pads rather than paper or online assessments. This method provides an immediate way of assessing knowledge and provides instant feedback to delegates. However, this may not suit all delegates as some may prefer to take more time to consider their responses. Assessment of knowledge at the end of the day reflects what has been learned but not what has been retained. An assessment after a time interval can determine whether the improvement in knowledge has been maintained. In our study, assessment results from a month after the course increased from assessment results on the day. Although this increase was not significant it shows that knowledge had been retained. Learning can also be reinforced by suggesting that delegates follow up with recommended reading after the course, although in our study only 58% of those who passed the post course assessment had read any of the recommended reading.

When planning and designing training, it is important to consider what will be the most suitable format to enable the group being taught to learn effectively. We designed our course to be delivered in one day in response to results of the UK dietitians survey [10]. Similar to other studies [14,16,17] we found that knowledge could be improved by designing and implementing training appropriate to the needs of our group following a needs assessment survey.

We have shown that delegates’ overall perceived level of knowledge and confidence improved occurring alongside actual increase in knowledge; an essential combination which ensures that correct advice can be given with confidence. Overall satisfaction with the day was high. However, discussions during the case studies at the end of the day and evaluations afterwards highlighted that there was still some confusion over the introduction of soya which may have impacted on confidence scores post asessment. It was also noted that the number of correct responses to one of the formulae questions improved between pre course and on the day answers (56% to 78%) but dropped again on the post course assessment (to 66%) suggesting that this area could have been covered more clearly. The course content has been amended to clarify these areas in more detail for future courses by altering slides and including more opportunity for discussion.

Dietitians valued the fact that the course provided more than a basic study day and that they would be awarded a certificate to show they had met the competencies if they achieved the required level in the assessments. This will provide a valuable addition to their CPD portfolios as well as improve quality of care to patients with CMPA. It may also help to raise the profile of dietitians with other health professionals.

### Limitations

In common with Springston et al. [14] and Yu et al. [16], our assessment questions included case scenarios in an attempt to ask delegates to use what they had learned in a fictional but practical example. This is a standard method of introducing the ‘student’ to examples of problems they may face in a real clinical setting. This method can also be used via online learning modules such as the WAO online food allergy module [15]. However, they are not an equal subsititute for advising a patient in person so the ability to put knowledge into practice in a clinical setting has not been evaluated. This could be considered as being the only way that competence can truly be measured but it is not practical for every dietitian or health professional to be monitored and tested in a real clinical setting at this level of training. Assessment by using questions relating to practical scenarios may be the closest alternative.

Although presentation content was determined by set objectives, the delivery of the presentations may vary between different speakers. In order to ensure that all key points are made, future course material will contain additional notes for speakers.

Although level of knowledge was shown to have been retained after one month, we have not assessed level of knowledge again at a later date. Future courses could reassess a year afterwards to measure ongoing retention of knowledge and find out how this has impacted on practice.

### Future use

The course package has been updated, further dates have been arranged for running more courses and is now available for rolling out. This package format could be used in other countries with only small adjustments made to allow for differences in available products in order to deliver training in CMPA. Courses in other areas of food allergy could be designed using the same format, thus improving competencies in a wider range of food allergies both in the UK and worldwide. As competencies are developed in other medical disciplines both in the UK and worldwide, this course format could also be used to deliver courses in other subjects.

## Conclusion

Following international guidelines on the diagnosis and management of food allergies and the international need for further training we have developed and implemented a training course for dietitians based on national competencies. The course provides a ‘step-up’ from standard one day courses. This format could be used to educate dietitians in other areas of food allergy as highlighted in the UK, US and Australian dietitians surveys [9,10,12] and will help to improve the standard of care to patients with food allergies in the UK and worldwide.
